# Angiopoietins in Diabetic Retinopathy: Current Understanding and Therapeutic Potential

**DOI:** 10.1155/2019/5140521

**Published:** 2019-08-14

**Authors:** Michael Whitehead, Andrew Osborne, Peter S. Widdowson, Patrick Yu-Wai-Man, Keith R. Martin

**Affiliations:** ^1^Van Geest Building, West Forvie Site, Addenbrookes Biomedical Campus, Cambridge CB2 0PY, UK; ^2^Cambridge Centre for Brain Repair, Department of Clinical Neurosciences, University of Cambridge, Cambridge, UK; ^3^Camburgh House 27 New Dover Road, Canterbury, Kent, CT1 3DN, UK; ^4^Ikarovec Ltd., Canterbury, UK; ^5^MRC Mitochondrial Biology Unit, Department of Clinical Neurosciences, University of Cambridge, Cambridge, UK; ^6^Cambridge Eye Unit, Addenbrooke's Hospital, Cambridge University Hospitals, Cambridge, UK; ^7^NIHR Biomedical Research Centre at Moorfields Eye Hospital and UCL Institute of Ophthalmology, London, UK; ^8^Wellcome Trust-MRC Cambridge Stem Cell Institute, University of Cambridge, UK; ^9^Centre for Eye Research Australia, Melbourne, Australia; ^10^University of Melbourne, Melbourne, Australia

## Abstract

Diabetic retinopathy (DR) is the commonest cause of blindness in the working-age population of the developed world. The molecular pathophysiology of DR is complex, and a complete spatiotemporal model of the disease is still being elucidated. Recently, a role for angiopoietin (Ang) proteins in the pathophysiology of DR has been proposed by several research groups, and several aspects of Ang signalling are being explored as novel therapeutic strategies. Here, we review the role of the Ang proteins in two important forms of DR, diabetic macular oedema and proliferative diabetic retinopathy. The function of the Ang proteins in regulating blood vessel permeability and neovascularisation is discussed, and we also evaluate recent preclinical and clinical studies highlighting the potential benefits of modulating Ang signalling as a treatment for DR.

## 1. Introduction to Diabetic Retinopathy

Diabetic retinopathy (DR) is the commonest cause of blindness in the working-age population of the developed world [1]. Around 90 million people are thought to be affected by DR, and this figure is expected to rise over the coming decades with the rapidly increasing prevalence of obesity worldwide and an ageing population with diabetes [1].

Two subgroups of DR account for most diabetic visual loss, namely, proliferative diabetic retinopathy (pDR) and diabetic macular oedema (DMO). In pDR, retinal neovascularisation is prominent and the onset of dysregulated angiogenesis is considered a hallmark feature [2]. In DMO, retinal vascular hyperpermeability leads to the leakage of blood plasma components into the retina [3]. In the early stages of DR, patients are often asymptomatic, but as the disease severity progresses over time, they frequently report visual disturbances such as blurred vision and in some cases, severe loss of vision due to complications including vitreous haemorrhage or tractional retinal detachment [4].

The molecular pathophysiology of DR is complex, and a detailed spatiotemporal model of the disease is still being elucidated. In patients with type I and type II diabetes mellitus (DM), poor glycaemic control leads to hyperglycaemia, which in turn drives aberrant regulation of at least five key biochemical pathways. These are the polyol pathway, the protein kinase C (PKC) pathway, advanced glycation end product (AGE) formation, the hexosamine pathway, and poly (ADP-ribose) polymerase upregulation [5]. Dysregulation of these pathways exacerbates oxidative stress, for example, with increased production of reactive oxygen species (ROS), which in turn leads to mitochondrial dysfunction, inflammation, and hypoxia. As a result, there is upregulation of vascular endothelial growth factor (VEGF), which has been implicated as a key causative factor of retinal neovascularisation and vascular hyperpermeability in pDR and DMO, respectively [5–8].

Currently, a number of treatments are available to clinicians for DR, including the optimisation of glycaemic control with regular injections of insulin, glucocorticoid therapy, PKC inhibitors, fenofibrate, laser photocoagulation, and in selected cases, vitreoretinal surgery [9–13]. More recently, anti-VEGF antibody treatments have become available, and these have demonstrated significant improvements in patient outcomes when compared to conventional therapeutic options [14].

Despite these advances, visual loss due to DR remains a major public health issue, and efforts are still underway to develop novel treatments for DR to address this unmet medical need. In recent years, a number of preclinical and clinical programmes have described the use of nonsteroidal anti-inflammatory drugs (NSAIDs), antibiotics, immunosuppressants, oxidative stress inhibitors, and vitriol viscosity inhibitors for treating DR [15–18]. Furthermore, the angiopoietin (Ang)/Tie2 signalling axis has emerged as a potential therapeutic strategy, and a number of clinical trials have demonstrated the efficacy of a number of pharmacologic and biologic mediators of the Ang/Tie2 pathway [19].

## 2. The Angiopoietin/Tie2 Signalling Axis

The Ang/Tie2 signalling axis is a key regulator of angiogenesis. Whilst the VEGF pathway is thought to be important for inducing endothelial cell sprouting and primary network formation, the Ang/Tie2 pathway regulates blood vessel remodelling and maturation in the later stages of the angiogenic process [20].

Whilst Ang1 and Ang4 are known agonists of Tie2 receptor activity, the role of Ang2 has been less certain [21, 22]. Based on more recent data, a consensus has emerged pointing towards a role for Ang2 as a negative modulator of Tie2 activity. Ang2 is also thought to act as a partial agonist/antagonist of Tie2 function by acting as a competitive inhibitor of Ang1 and Ang4 binding [23–25].

All Ang proteins are secreted factors that bind to the Tie2 receptor. The Tie2 receptor is highly expressed on endothelial cells, and it is composed of immunoglobulin-like domains, epidermal growth factor-like domains, and fibronectin type III domains [26]. Following activation, Tie2 demonstrates strong kinase activity and becomes phosphorylated on several cytoplasmic tyrosine residues. This results in the downstream activation of a number of pathways, including the PI3-kinase/protein kinase B (AKT) and extracellular signal-regulated kinase (ERK) pathways, which inhibit de novo blood vessel growth and vascular hyperpermeability [23].

Whilst a structural characterisation of the Ang4 protein is lacking, efforts have been made to resolve the structure of the Ang1/Tie2 and Ang2/Tie2 ligand/receptor interactions. X-ray crystallography analysis of Ang1/Tie2 interactions, coupled with structure-based mutagenesis, has been used to identify molecular surfaces necessary for Tie2 activation. The Ang fibrinogen-like domain has been shown to mediate the Tie2 agonistic properties of Ang1, and when this domain was transferred into Ang2, the protein developed constitutive Tie2 phosphorylating capabilities [27, 28].

## 3. Pathological Consequences of Angiopoietin Dysregulation

The Ang proteins regulate a large number of biochemical pathways and physiological processes, and as such, they have been implicated in a number of pathological pathways across a broad range of diseases. Ang dysregulation has been implicated in diabetic nephropathy, with aberrant Ang function correlating with abnormal glomerular barrier filtration [29]. The role of Ang proteins in diabetic wound healing is also an area of active research. In a streptozotocin- (STZ-) induced mouse model of diabetes, Ang1 treatment was shown to upregulate matrix metalloproteinase- (MMP-) 9 and stem cell factor levels which were associated with improved reepithelialisation, neovascularisation, and endothelial progenitor cell recruitment [30]. An Ang-based peptide mimetic, Vasculotide (Vasomune), was shown to promote endothelial cell survival, migration, and MMP-2 synthesis in a skin-wound model employing the *db*/*db* transgenic diabetic mouse model. Decreased wound closure times and a significant increase in granulation tissue were also reported [31].

In the lymphatic system, Ang1/Tie2 signalling was found to attenuate oedema formation, and decreased inflammation was seen in Ang1-overexpressing mice exposed to UVB irradiation [32]. In the nervous system, Ang1 induced neurite outgrowth in PC12 cells at levels comparable with nerve growth factor (NGF). Interestingly, this effect was shown to be *β*1-integrin-dependent, but Tie2-independent, demonstrating that Ang1 is capable of binding to multiple receptors [33].

The Ang proteins have been closely studied in oncological models given their close association with angiogenic processes. In a study looking at the role of Ang2 in the migration of glioma tumour cells, Ang2 was found to bind to *α*5*β*1-integrins on Tie2 receptor-negative cells via a specific residue, Gln^362^, and this in turn upregulated focal adhesion kinase (FAK). This Ang2/*α*5*β*1-integrin interaction was shown to enhance the migration and invasion of the glioma cells, suggesting an important role for Ang2 in mediating tumour growth and metastasis [34].

## 4. Role of Angiopoietins in Diabetic Retinopathy

It has become increasingly clear that Ang proteins regulate a number of physiological systems, and dysregulation of these pathways has important pathological consequences. This family of proteins has been implicated in DR as mediators of the permeability of the blood-retinal barrier (BRB) and in the regulation of pericyte function, angiogenesis, and apoptosis. Various treatment modalities used in DR are thought to alter Ang protein levels, and the ability to specifically modulate Ang protein function therefore represents a promising therapeutic strategy.

### 4.1. Ang Proteins as Risk Factors for Diabetic Retinopathy

The levels of Ang1 and Ang2 proteins have been determined in the eyes of patients with pDR and DMO, but there is little consensus in the literature whether these levels are independent risk factors for the development of these diabetic complications. In most reports, Ang2 concentrations have been reported to be increased in patients with both pDR and DMO, implying a possible role in neovascularisation and vascular hyperpermeability through attenuation of Tie2 activity [35–37]. However, one study reported lower levels of Ang2 in pDR patients, an apparent inconsistency that could reflect the established nature of the neovascularisation seen in the group of patients studied [38].

Ang1 has been reported to be upregulated in DMO, but there are conflicting data with regard to whether Ang1 is upregulated in pDR [35, 36]. These observations are perhaps unexpected given that a proposed function of Ang1 is to prevent neovascularisation and vascular hyperpermeability. Further research is needed to define the relative ratios of Ang1 and Ang2 levels in the eyes of patients with different diabetic complications and their pathophysiological relevance.

### 4.2. Role of the Ang Proteins in Regulating Blood-Retinal Barrier Function and Vascular Permeability in DR

**Figure 1:**
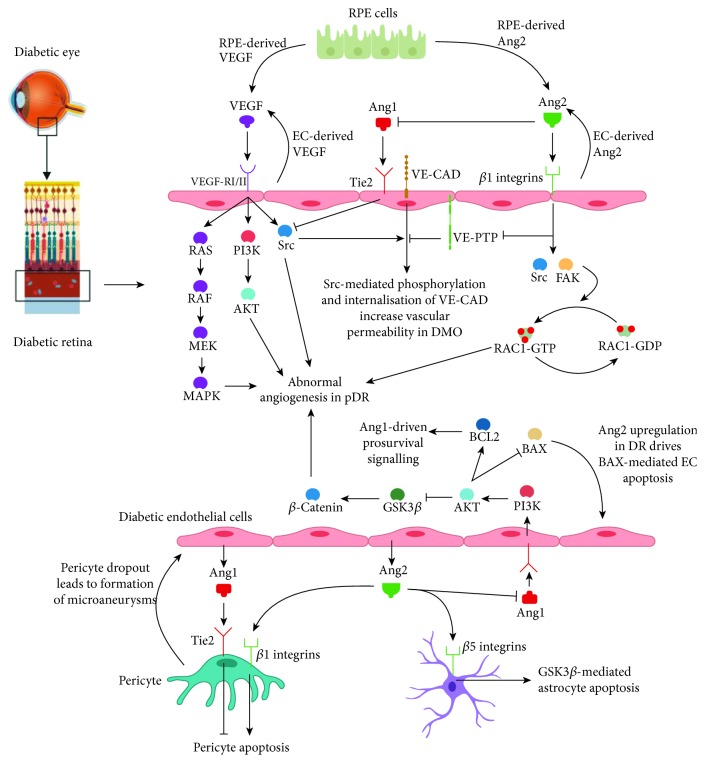
Overview of Ang/Tie2 signalling in the diabetic eye, including crosstalk with the VEGF pathway. EC = endothelial cell; VE-CAD = VE-cadherin.

It is now well established that Ang1 can induce vascular remodelling by upregulating highly organised angiogenic processes and by facilitating the tightening of endothelial cell junctions [39]. In contrast to other angiogenic molecules like VEGF, neovascularisation in the presence of Ang1 is highly ordered and hierarchical, and Ang1 has been shown to rescue the formation of a poorly remodelled and abnormally permeable vascular network in a murine model of diabetic retinopathy [40]. The exact mechanisms involved remain unclear, but the effect of Ang1 could be antagonistic to that of VEGF-induced vascular permeability. VEGF acts via Src to induce vascular endothelial cadherin (VE-cadherin) tyrosine phosphorylation and subsequent internalisation, which results in elevated endothelial cell permeability (Figure 1) [41]. By sequestering Src, Ang1/Tie2 interactions could inhibit this VEGF-induced effect and prevent endothelial cell permeability [42]. In addition to Src regulation, Ang1 is also thought to influence vascular endothelial phosphotyrosine phosphatase (VE-PTP) activity. The current evidence points towards Ang1 promoting the formation of Tie2/VE-PTP/VE-cadherin complexes at the cellular membrane, thereby preventing the dissociation of VE-PTP and VE-cadherin, which normally increases vascular permeability via VE-PTP-mediated VE-cadherin dephosphorylation and internalisation (Figure 1) [43].

The role of Ang2 in regulating BRB function and vascular permeability has been explored in murine models of DR. The induction of diabetes in rats (with an STZ injection) and human retinal endothelial cells (with 5-30 mM concentrations of glucose) was found to increase Ang2 mRNA levels with an associated decrease in VE-cadherin levels (Figure 1) [44]. Methylglyoxal is a product of the hyperglycaemia-induced AGE pathway, and increased Ang2 expression in the retinal pigment epithelium (RPE) was linked with abnormal microvascular permeability via increased methylglyoxal synthesis [45]. Elevated Ang2 synthesis was also correlated with increased levels of proapoptotic BAX and decreased levels of antiapoptotic BCL-2, indicating an important role in endothelial cell dysfunction and apoptosis in DR [45]. Interestingly, Ang2 has been shown to activate *β*1-integrins in order to promote vascular destabilisation via the regulation of VE-cadherin-containing cell-cell junctions (Figure 1) [46].

### 4.3. Role of the Angiopoietins in Angiogenesis in DR

The Ang/Tie2 signalling axis modulates the survival and migration of endothelial cells and is a key regulator of both vascular remodelling and the maintenance of vascular integrity [47]. Unsurprisingly, the role of Ang proteins in facilitating angiogenesis has been implicated in a number of ocular pathologies, including age-related macular degeneration, neovascular glaucoma, and trachoma [48].

In the context of diabetic ocular pathology, Ang1 and Ang2 were found to enhance the effects of VEGF-mediated angiogenesis when assessed *in vitro* with a bovine retinal capillary endothelial cell line-based tube formation assay [49]. However, the assumption that these two proteins upregulate aberrant angiogenesis in pDR has recently been called into question. Ang1 supplementation in an oxygen-induced *in vivo* model of diabetic retinopathy blocked the development of retinal disease by inhibiting aberrant angiogenesis and vascular leakage [50]. This observation was replicated in other biological systems strongly indicating that the functions of Ang1 and Ang2 are dualistic [23, 51, 52]. It has been proposed that Ang2 facilitates disease progression in DR. Overexpression of Ang2 in transgenic mouse models demonstrated reduced pericyte capillary coverage and increased intraretinal neovascularisation via the inhibition of Ang1/Tie2 interactions (Figure 1). Furthermore, Ang2 upregulation was found to increase VEGF expression, which is a key factor in the development and progression of diabetic angiogenesis [53]. Recent evidence points towards Ang2 differentially regulating angiogenesis through Tie2 and integrin signalling. Angiogenesis-activated endothelial cells were found to harbour a subpopulation of Tie2-negative integrin-overexpressing cells, and Ang2 was bound to these integrins, inducing angiogenesis in a FAK- and RAC-1-dependent manner (Figure 1) [54].

### 4.4. Role of Angiopoietins in Inducing Apoptosis in DR

Ang2 upregulation has been correlated with reduced pericyte coverage and increased pericyte apoptosis (REFS). In a diabetic rat model, Ang2 was upregulated almost 30-fold compared with normal controls, and this upregulation preceded pericyte loss. Injection of recombinant Ang2 into the eyes of normal rats also resulted in pericyte loss in a dose-dependent manner [55].

In retinal pericytes, Ang1 and Ang2 had opposite roles depending on the culture conditions. In the presence of tumour necrosis factor alpha (TNF*α*), Ang1 protected pericytes against apoptosis whereas Ang2 accelerated the onset of programmed cell death [56]. In mouse models of DR, increased levels of Ang2 correlated with elevated loss of pericytes, and vascular endothelial cells were implicated as a potential source of Ang2. The latter was also found to induce apoptosis under high glucose conditions and via the p53 pathway by binding to *α*3*β*1-integrins. It is therefore likely that integrin binding is the mechanism by which Ang2 induces pericyte apoptosis (Figure 1) [57].

Upregulation of Ang2 enhances vascular damage during hyperglycaemia in transgenic models of diabetic retinopathy. Overexpression of Ang2 in nondiabetic and STZ-induced diabetic mice significantly worsened the underlying vascular pathology when pericyte apoptosis and acellular capillary formation were assessed (Figure 1) [58]. These findings have been corroborated in diabetic models investigating the effect of nucleoside diphosphate kinase B (NDPKB) deficiency [59]. NDPKB deficiency upregulated Ang2 expression and protein N-acetylglucosamine modification, which is a product of the hexosamine pathway, and this was associated with decreased pericyte coverage and elevated acellular capillary formation [59].

Ang2 could also induce astrocyte apoptosis via an integrin-dependent mechanism. DR was induced in STZ mouse models, and elevated Ang2 levels were correlated with astrocyte loss. Interestingly, Ang2-mediated astrocyte apoptosis could be inhibited with an anti-Ang2 neutralising antibody, and this likely occurred via a GSK3*β*-dependent mechanism. Activation of GSK3*β* seemed to occur downstream of Ang2 binding to *α*v*β*5-integrins, and *in vivo* injections of an anti-*α*v*β*5-integrin antibody were sufficient to inhibit astrocyte apoptosis in these particular STZ mouse models (Figure 1) [60].

## 5. Role of Angiopoietin/Tie2 Signalling as Novel Therapeutic Strategies

The modulation of Ang1 and Ang2 levels is an attractive therapeutic target for DR [61]. Administration of Ang1 protects the vasculature against VEGF-induced leakage in a number of physiological systems [62]. Overexpression of Ang1 in a transgenic diabetic mouse model inhibited the onset and progression of oxygen-induced neovascularisation [63]. Ang1 also blocked VEGF-induced leakage of mannitol from the systemic circulation into the retina (Table 1). In a VEGF-overexpressing transgenic mouse model, Ang1 supplementation prevented exudative retinal detachment, but it did not have any effect on established retinal neovascularisation [64]. Consistent with these findings, intravitreal injections of adenovirus expressing Ang1 prevented leukocyte adhesion, apoptosis of retinal endothelial cells, and subsequent breakdown of the BRB in an STZ-induced rat model of DR [65].

Although Ang1 supplementation represents a viable treatment option for DR, the instability and insolubility of the Ang1 protein represent major technical challenges. Pharmacological research is now being directed towards creating more stable and soluble forms of Ang1 with more potent activity. Cartilage oligomeric matrix protein- (COMP-) Ang1 was shown to attenuate the structural and functional hallmarks of DR in a transgenic mouse model [66]. When delivered into the retina using an adeno-associated virus serotype 2 (AAV2) viral vector, leukocyte adhesion and vascular permeability were decreased and retinal neurophysiological responses were improved to levels similar to that of nondiabetic control animals. AAV2.COMP-Ang1 was also shown to enhance the therapeutic benefit of the intravitreal delivery of endothelial colony-forming cells by facilitating their integration into the retinal vasculature (Table 1) [66].

In order to enhance Tie2 activity with Ang1 supplementation, another strategy that is being explored is the inhibition of VE-PTP using biological and pharmacologic modalities. Similarly, incubation of Tie2 expressing endothelial cells in culture with AKB-9778, a small molecule inhibitor of VE-PTP, resulted in increased Tie2 phosphorylation [67, 68]. Crucially, inhibition of VE-PTP has been shown to prevent ischaemia-induced retinal neovascularisation and choroidal neovascularisation. When used in combination with the anti-VEGF agent Aflibercept, an additive effect was seen in the reduction of vascular leakage in VEGF-overexpressing transgenic mouse models (Table 2) [68]. Cumulative experimental data supports the translational potential of VE-PTP inhibition in the treatment of sight-threatening pDR and DMO. In a phase IIb randomised clinical trial (TIME-IIb) that recruited 144 patients with DMO, patients treated with AKB-9778 showed a greater reduction in central subfield thickness (CST) compared with the anti-VEGF (ranibizumab) monotherapy group, but this structural benefit did not result in a significantly better visual outcome (Table 2) [69–73]. One possible reason why AKB-9778 failed to improve visual acuity in the DMO patients may be related to the absolute dependence of Ang1 to activate Tie2 receptors and the potential for significant loss in agonist concentrations through pericyte death in this disease.

The inhibition of Ang2 as a means of elevating Tie2 phosphorylation and promoting vascular stabilisation has been investigated in two clinical trials for DMO. In the phase II RUBY trial, patients with DMO received high and low doses of an anti-Ang2 antibody in combination with Aflibercept (REGN910-3) or Aflibercept on its own. No additional visual benefit was observed in the dual treatment group (Ang2 and VEGF inhibition) compared with the Aflibercept monotherapy group (Table 2) [72]. The BOULEVARD phase II clinical trial investigated a bispecific antibody (RG-7716) targeting both VEGF and Ang2 proteins. At week 24, patients receiving this experimental treatment modality showed a statistically superior gain in visual acuity compared with those receiving ranibizumab. However, it should be noted that the BOULEVARD trial compared RG-7716 against low-dose (0.3 mg) ranibizumab and not Aflibercept, which is now considered the gold standard for the treatment of DMO. Furthermore, the absolute visual benefit was relatively modest, suggesting that a head-to-head comparison of RG-7716 with Aflibercept might not demonstrate the superiority of dual VEGF and Ang2 inhibition over conventional treatment (Table 2) [73]. Despite only modest benefits of Ang2 neutralisation, no clinical studies have yet examined the benefits of elevating levels of Tie2 agonists, both to counter loss in pericyte-derived Ang1 and to counterbalance increased Ang2 levels.

Several preclinical studies have demonstrated the therapeutic benefit of modulating Tie2 signalling in DR. Although exciting, the translational potential of this particular pathway remains to be demonstrated, and further research into Ang/Tie2 is needed to identify other targets that could be manipulated either pharmacologically or with gene therapy approaches.

## 6. Conclusion

The known reported effects of Ang proteins on vascular function strongly suggest a key role in DR pathophysiology. Multiple studies have demonstrated that interaction of Ang proteins with the Tie2 receptor serves to mediate a number of intracellular signalling pathways in a number of cell types, including endothelial cells, RPE cells, and a number of neuronal cell types, all of which are affected to some degree in DR. The binding of the Ang proteins to Tie2 influences a range of physiological events, including blood vessel permeability and neovascularisation. In DMO, dysregulated Ang signalling is thought to mediate destabilisation of the blood-retinal barrier, whilst in pDR, it has been shown to induce pathological neovascularisation in the retina. Preclinical and recent clinical studies outlining the promising therapeutic potential of mediating Ang/Tie2 signalling have started to confirm Tie2 receptors as a therapeutic target for DR although no agents which directly stimulate the receptor have so far been examined. In summary, Ang proteins play a vital role in DR and are exciting therapeutic candidates towards the development of new treatments for the condition.

## Figures and Tables

**Table 1:** Overview of preclinical trials assessing the efficacy of Tie2 signalling for the treatment of DR.

Reference	Therapy	Therapeutic mechanism	Animal model	Inhibited angiogenesis?	Inhibited vasopermeability?	Comments
Joussen et al. [65]	IVT rAng1, Ad-Ang1	Upregulated Tie2 phosphorylation	STZ mouse	n/a	Yes	Also showed Ang1 protects against leukocyte-mediated EC damage
Nambu et al. [63]	Overexpression of Ang1 using transgenic model	Upregulated Tie2 phosphorylation	OIR mouse, laser-induced NV mouse	Yes	Yes	
Nambu et al. [64]	Overexpression of Ang1 using transgenic model	Upregulated Tie2 phosphorylation	OIR mouse, laser-induced NV mouse	No	n/a	Prevented retinal detachment
Lee et al. [50]	IVT of rAng1	*β*5 integrin signalling	OIR mouse	Yes	Yes	Also shown to be effective in ROP
Shen et al. [68]	IVT of anti-Ang2 antibody	Upregulated Tie2 phosphorylation	OIR mouse	Yes	Yes	
Cahoon et al. [66]	AAV2.COMP-Ang1	Upregulated Tie2 phosphorylation	InsIIAkita transgenic mouse	n/a	Yes	Also restored retinal neurophysiological responses

IVT = intravitreal injection; OIR = oxygen-induced retinopathy; STZ = streptozotocin; EC = endothelial cell; NV = neovascularisation; ROP = retinopathy of prematurity; InsIIAkita = mouse model of diabetes with mutated insulin II gene.

**Table 2:** Overview of agents targeting the Ang/Tie2 pathway that are currently in clinical development.

Drug name	Developer	Target	Trial phase	Indication	Patients enrolled	Control	Primary outcome measure	Result
AKB-9778	Akebia Therapeutics	VE-PTP	IIb	DMO	167	Placebo	DRSS	Failed
REGN910-3	Regeneron	Ang2 and VEGF	II	DMO	301	Aflibercept	BCVA	Failed
RG-7716	Roche Genentech	Ang2 and VEGF	II	DMO	230	Low-dose ranibizumab	BCVA	Success

DRSS = diabetic retinopathy severity score; BCVA = best-corrected visual acuity.
